# Effects of High-Fat Diet on eHSP72 and Extra-to-Intracellular HSP70 Levels in Mice Submitted to Exercise under Exposure to Fine Particulate Matter

**DOI:** 10.1155/2019/4858740

**Published:** 2019-01-06

**Authors:** Iberê Machado Kostrycki, Guilherme Wildner, Yohanna Hannah Donato, Analú Bender dos Santos, Lílian Corrêa Costa Beber, Matias Nunes Frizzo, Mirna Stela Ludwig, Kevin Noel Keane, Vinicius Cruzat, Cláudia Ramos Rhoden, Thiago Gomes Heck

**Affiliations:** ^1^Research Group in Physiology, Department of Life Sciences, Regional University of Northwestern Rio Grande do Sul State (UNIJUI), Ijuí, RS, Brazil; ^2^Laboratory of Oxidative Stress and Air Pollution, Postgraduate Program in Health Sciences, Federal University of Health Sciences of Porto Alegre (UFCSPA), Porto Alegre, RS, Brazil; ^3^Postgraduate Program in Integral Attention to Health (PPGAIS-UNIJUÍ/UNICRUZ), Ijuí, RS, Brazil; ^4^School of Pharmacy and Biomedical Sciences, Curtin Health Innovation Research Institute, Curtin University, Perth 6102, Australia; ^5^Faculty of Health, Torrens University Australia, Melbourne, Victoria 3065, Australia

## Abstract

Obesity, air pollution, and exercise induce alterations in the heat shock response (HSR), in both intracellular 70 kDa heat shock proteins (iHSP70) and the plasmatic extracellular form (eHSP72). Extra-to-intracellular HSP70 ratio (H-index = eHSP70/iHSP70 ratio) represents a candidate biomarker of subclinical health status. This study investigated the effects of moderate- and high-intensity exercise in the HSR and oxidative stress parameters, in obese mice exposed to fine particulate matter (PM_2.5_). Thirty-day-old male isogenic B6_129_F_2_/J mice were maintained for 16 weeks on standard chow or high-fat diet (HFD). Then, mice were exposed to either saline or 50 *μ*g of PM_2.5_ by intranasal instillation and subsequently maintained at rest or subjected to moderate- or high-intensity swimming exercise. HFD mice exhibited high adiposity and glucose intolerance at week 16th. HFD mice submitted to moderate- or high-intensity exercise were not able to complete the exercise session and showed lower levels of eHSP70 and H-index, when compared to controls. PM_2.5_ exposure modified the glycaemic response to exercise and modified hematological responses in HFD mice. Our study suggests that obesity is a critical health condition for exercise prescription under PM_2.5_ exposure.

## 1. Introduction

Obesity is an increasing worldwide issue and is associated with comorbidities, such as insulin resistance, dyslipidemia, hypertension, cancer, and cardiovascular disease [1]. Overnutrition and physical inactivity are the primary factors that contribute to the burden of obesity. Interestingly, in animal models of obesity [2] and also in humans [3] exposed to a high-caloric intake diet, physical exercise can attenuate the effects of diet-induced obesity. Conversely, the importance of exercise continues to be undervalued despite evidence of its protective effects. It is estimated that >30% of the global adult population does not meet the minimum levels of daily exercise and can be defined as physically inactive [4]. The metabolic alterations resulting from this sedentary lifestyle, along with overnutrition include an increased abdominal and visceral adiposity, which significantly contributes to insulin resistance chronic low-grade inflammation [5] and oxidative stress [6, 7].

Obesity and associated comorbidities are more prevalent in urban areas, where individuals are also exposed to another major global health problem, the air pollution exposure, mainly represented by a fine particulate matter (PM_2.5_) [8, 9]. PM_2.5_ is formed from the combustion processes, including vehicles, power plants, and burning related to agricultural or industrial work. As PM_2.5_ is inhaled, it invades the respiratory tract and the vascular system [10] promoting oxidative stress in tissues such as the lungs and heart which has been associated with the development of pulmonary inflammation [11, 12]. In overweight and obese individuals, PM_2.5_ exposure has been strongly associated with the risk of cardiovascular disease, stroke, and insulin resistance, which is potentiated by the rising inflammatory effects of adiposity, increased BMI and increased waist-to-hip ratio [9, 13–15].

At the most basic level, mammalian cells have developed a range of adaptations to survive and respond to these different types of hostile situations such as heat shock, oxidative stress, and inflammation, by changing the expression of heat shock proteins (HSPs). HSPs are a family of polypeptides clustered according to their molecular weight and have many intracellular functions. The most important is to act as a molecular chaperone and prevent inappropriate protein interactions and degradation. Also, recent studies have demonstrated that the intracellular 70 kDa heat shock protein (iHSP70) can act as an important anti-inflammatory agent particularly in stressful cellular situations [16, 17]. Conversely, HSP70 can be released into the extracellular space (i.e., eHSP70), where it functions as a stress signal and a proinflammatory molecule [18–20]. For example, the chronic exposure to PM_2.5_ increases plasma levels of eHSP70 which can contribute to vascular dysfunction and the increased susceptibility to cardiovascular disease [21, 22]. In obesity and type 2 diabetes, eHSP70 has been negatively correlated with iHSP70 in skeletal muscles and led to impaired glucose handling. Indeed, elevated levels of eHSP70 are associated with insulin resistance and beta cell failure in elderly volunteers [18].

Although the precise regulation of extra-to-intracellular HSP70 ratio (H-index = eHSP70/iHSP70) is still unknown [15, 23], exercise is a potent mediator of the heat shock response (HSR) [17], when under exposure to PM_2.5_ [24]. Thus, in the present study, we aimed to investigate the effects of high-fat diet consumption on eHSP70 and H-index, in mice submitted to acute moderate- and high-intensity exercise, following exposure to PM_2.5_. The impact of these conditions on oxidative, glycaemic, and hematological parameters was also assessed.

## 2. Material and Methods

### 2.1. Animals, Diet, and Experimental Design

**Figure 1:**
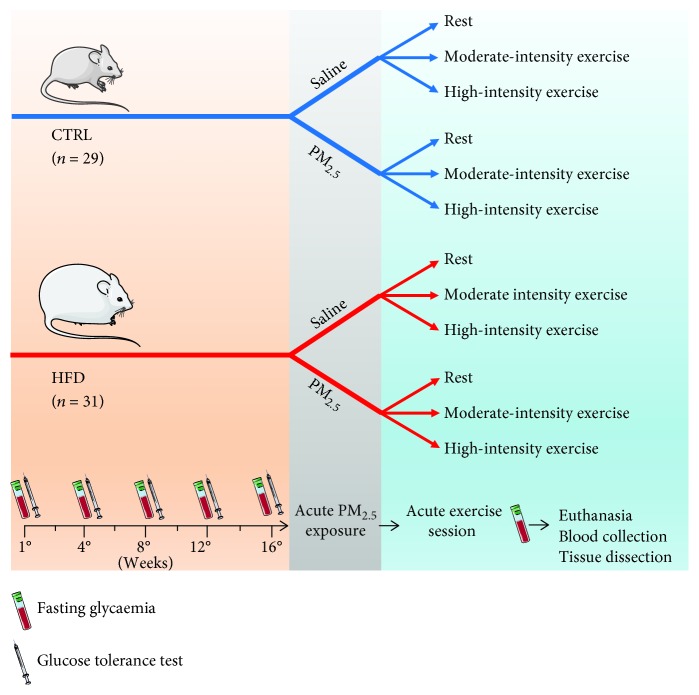
Experimental design. Thirty-day-old male mice were randomly housed in two groups: mice receiving standard chow (CTRL, *n* = 29) or high-fat diet (HFD, *n* = 31) for 16 weeks. After 16 weeks, CTRL and HFD mice were randomly subdivided and exposed to a nasotropic instillation of PM_2.5_ or saline solution, then rested, or subjected to moderate- or high-intensity physical exercise. The mice were euthanized by decapitation, and blood and lungs were collected for biochemical analyses.

Thirty-day-old male isogenic B6_129_F_2_/J mice weighing about 18 g were obtained from the Animal Facility of the Regional University of Northwestern of Rio Grande do Sul State (UNIJUÍ) and maintained in semimetabolic cages on a 12 h light/dark cycle (lights on at 07:00) and at a room temperature of 22 ± 2°C with 60% relative humidity. The animals were randomly housed in two groups: mice receiving standard laboratory mouse chow (CTRL, *n* = 29) or those on a high-fat diet (HFD, *n* = 31) for 16 weeks. All animals had free access to water and were ad libitum fed with CTRL or HFD chow. Animals from the CTRL group received a pelleted diet, consisting of crude protein, fibrous matter, and minerals (provided by NUVILAB CR1, NuVital Nutrients, Curitiba, Brazil) with a total energy of 16.6 MJ/kg and specifically included 11.4% fats, 62.8% carbohydrates, and 25.8% proteins. The HFD group received a lard-based diet (37.4% *w*/*w*) with a total energy of 22.8 MJ/kg and specifically included 58.3% fats, 24.5% carbohydrates, and 17.2% proteins (Bock et al. 2015; Goettems-Fiorin et al. [15]). After 16 weeks, CTRL and HFD mice were randomly subdivided and exposed to a nasotropic instillation of PM_2.5_ or saline solution and were rested or submitted to moderate- or high-intensity physical exercise. The mice were euthanized by decapitation, and blood was collected for the determination of eHSP70 concentration in plasma. Also, the lungs were dissected for oxidative stress determination and the expression of iHSP70, allowing the calculation of the H-index. Epididymal white adipose tissue (WAT) was removed for adiposity analysis, and blood was collected for hematological analysis. The detailed experimental design and flow diagram is provided in Figure 1. All the procedures were approved by the Animal Ethics Committee of UNIJUÍ (CEUA 011/13), according to the guidelines of the Brazilian College on Animal Experimentation.

### 2.2. Characterization and Exposure to PM_2.5_

The pollutant PM_2.5_ was collected in a polycarbonate filter through a gravimetric collector on the terrace of the Faculty of Medicine, University of São Paulo (USP), in São Paulo, Brazil, as previously described [25]. The exposure site was located close to a monitoring station of the State of São Paulo Sanitation Agency. It is estimated that at least 100000 vehicles circulate daily on the main and lateral streets (~83% cars, ~10% diesel vehicles, and ~6% motorcycles). There are no industries or significant biomass sources in the surrounding area. PM_2.5_ trace element content was determined by neutron activation analysis, and their concentrations were as follows: arsenic = 12.91 ± 0.53; bromine = 8.88 ± 0.39; cobalt = 1.14 ± 0.04; iron = 1.15 ± 0.03; lanthanum = 2.33 ± 0.29; manganese = 27.5 ± 2.2; antimony = 8.73 ± 0.08; scandium = 0.141 ± 0.009; and thorium = 0.351 ± 0.50 (all expressed as ng·m^−3^ of air). Likewise, the PM_2.5_ sulphur concentration, determined by X-ray fluorescence analysis, was 1.424 ± 0.08 *μ*m·m^−3^. Almost all particles had a diameter of less than 10 *μ*m (1.2 ± 2.18 *μ*m), and about 98% of particles had a diameter of less than 2.5 *μ*m. Briefly, after exposure (24 h), the filter was removed and retained. Particles were obtained by sonication, with an ultrasound bath in seven sessions (50 min each) and suspended in saline solution at a concentration of 50 *μ*g of PM_2.5_ in 10 *μ*L of saline.

The nasotropic instillation of PM_2.5_ occurred immediately before the exercise session with a 10 *μ*L dose of the solution in the nostril of the animal. This procedure induced an apnoea reflex which promoted inhalation of the pollutant. Before instillation procedure, the particle suspension was submitted to a new sonication process for 10 minutes in a water bath ultrasound and was mixed 0 seconds in a vortex and, thus, immediately administrated. The intranasal dose of 50 *μ*g represents a high exposure to the particle and is equivalent to an urban environment exposure of approximately 50 *μ*g/m^3^.

### 2.3. Moderate- and High-Intensity Exercise Protocols

All animals were allowed to acclimatize to the water environment before the exercise protocol to avoid any stress response related to the new environment and situation. The adaptation period consisted of 8 min in individual swimming pool chambers (10 cm × 10 cm × 30 cm) filled with water (20 cm depth) at 31 ± 1°C for three consecutive days and without any weight attached to the tail. Individual swimming pool chambers with 20 cm of water prevented jumping and diving behavior and allowed energy expenditure higher than three metabolic equivalents (METs) [26]. In the subsequent week, animals were randomly assigned to each exercise intensity protocol for 20 minutes or the swimming time was recorded until the animals were fatigued (8 seconds below water surface) due to the exercise burden imposed by weight (i.e., 4% or 8% of body weight) attached to the base of the tail. All experiments were carried out between 1:00 and 3:00 p.m., and the room temperature was kept at 24°C. Sedentary animals (CTRL, HFD, PM_2.5_, and HFD + PM_2.5_ groups) remained at rest in shallow water. All the procedures were in accordance with those prescribed in The American Physiological Society's Resource Book for the Design of Animal Exercise Protocols [26], and an experienced researcher was present at all times to prevent drowning. The exercise intensity was evaluated by caudal venous lactate concentrations (~25 *μ*L of blood) using a lactate analyzer (Accutrend® Plus System, Roche Diagnostics®, Indiana, USA.).

### 2.4. Measurement of Plasma Glucose, GTT, and Adiposity

Throughout the 16 weeks, the total body weight, fasting glycaemia, and glucose tolerance test (GTT) were evaluated at the 30th day and at the 4th, 8th, 12th, and 16th week. Blood glucose concentrations were measured at rest, before and after exercise using an Optium Xceed glucometer (Abbott Diabetes Care, Alameda, USA). For the GTT, food was withdrawn 12 h before the test and glycaemia was measured immediately before and at 30 and 120 min post glucose (1 g/kg in saline solution, i.p.) administration. The glycaemic response during the GTT was evaluated by IAUC method.

Total body adiposity was measured at the end of the 16th week to better characterize the installation of obesity. At the end of the experiment, adiposity was evaluated by dissecting and weighing WAT buying an analytical balance and the adiposity was expressed as a percentage of total body weight (WAT weight/total body weight).

### 2.5. eHSP70 Concentration in Plasma

Animals were euthanized immediately after the exercise session, and blood was collected in EDTA-treated tubes. The samples were then centrifuged at 2000 × *g*, at room temperature for 15 min to obtain plasma samples. The HSP70 plasma concentration (eHSP70) was measured by using a high-sensitivity HSPA1A-specific HSP70 ELISA Kit (Enzo Life Sciences, EKS-715, Farmingdale, USA) in diluted (1 :  4) plasma samples as recommended by the manufacturer. Absorbance was measured at 450 nm using a microplate reader (Mindray MR-96A) and a standard curve constructed from known dilutions of recombinant 72 kDa heat shock protein (HSP72) to allow quantitative assessment of eHSP70 plasma concentration. The intra-assay coefficient of variation was identified as being <2%. Although there are at least two isoforms of HSP70 (the 72 and 73 kDa HSPs, which are well known as the HSPA1A-inducible form and the cognate HSPA8 constitutive form, respectively), the levels of HSPA1A (eHSP72) can be used as representative of total eHSP70 secretion [23]. It is expected that both the inducible and constitutive forms should be delivered into the extracellular space after stressful conditions as acute exercise; however, eHSP72 has been used as biomarker of stress situations in previous studies related to particulate matter pollution, diabetes, and exercise [15, 17, 24]. Also, only HSPA1A ELISA kits have been sufficiently tested worldwide and are accepted to possess enough sensitivity (pg·mL−1 range) to detect minute quantities of HSP70 in plasma.

### 2.6. iHSP70, TBARS, and SOD in the Lungs

The lungs were dissected and freeze clamped in liquid nitrogen for further homogenization and analysis of the iHSP70 levels, the antioxidant activity of total superoxide dismutase (SOD), and thiobarbituric acid reactive substances (TBARS). iHSP70 expression was evaluated in the lungs by immunoblot analyses. Equivalent amounts of protein from each sample (∼40 *μ*g) were mixed with Laemmli's gel loading buffer (50 mM Tris, 10% (*w*/*v*) SDS, 10% (*v*/*v*) glycerol, 10% (*v*/*v*) 2-mercaptoethanol, and 2 mg/mL bromophenol blue) in a ratio of 1 : 1, boiled for 5 min, and electrophoresed in a 10% polyacrylamide gel (5 h in 15 mA/gel). After the proteins were transferred onto a nitrocellulose membrane (GE Healthcare) by electrotransfer (1 h in 100 V), the subsequently transferred bands were visualized with 3% (*w*/*v*) Red Ponceau S (Sigma-Aldrich). The procedures were performed with the SNAP i.d. (Merck Millipore) vacuum system for rapid immunoblotting. Membranes were washed with water and then blocked with 0.5% (*w*/*v*) fat-free milk buffer (TEN-Tween 20 solution (0.1%, *w*/*v*); TEN is 50 mM Tris, 5 mM EDTA, 150 mM NaCl, and pH 7.4). Membranes were then washed three times with wash buffer and incubated for 15 min with the monoclonal anti-HSP70 antibody (Sigma-Aldrich H5147, 1 : 1000). After three consecutive washes with wash buffer, peroxidase-labelled rabbit anti-mouse IgG (Sigma-Aldrich A9044) was utilized as a secondary antibody, at 1 : 15000 dilution. For gel loading control, Coomassie Blue staining (0.1% Coomassie Blue, 40% methanol, and 10% acetic acid) was used. Blot visualization was performed using ECL Prime Western Blotting Reagent (GE Healthcare). Quantification of bands was determined using the ImageJ® software. The data was presented in arbitrary units of iHSP70, normalized by *β*-actin expression.

For TBARS and total SOD analysis, a portion of the lung tissue was homogenized in potassium phosphate buffer at pH 7.4 containing the protease inhibitor PMSF (phenylmethylsulfonyl fluoride, 100 *μ*M). Afterwards, the homogenates were centrifuged at 1200 × *g* for 10 min at room temperature and the supernatant fractions were saved for protein determination by a spectrophotometric method [27] at 595 nm, using bovine serum albumin as standard. Homogenates were precipitated with 10% trichloroacetic acid (3 : 1 *v*/*v*), centrifuged, and incubated with 0.67% thiobarbituric acid (1 : 1 *v*/*v*) (T5500, Sigma) for 15 min at 100°C. The absorbance was measured at 535 nm. The amount of TBARS formed was expressed in nanomoles of malondialdehyde per milligram of protein (nmol MDA·mg prot^−1^). The MDA standard was prepared from 1,1,3,3,-tetramethoxypropane (Fluka, USA). Total SOD activity was determined by inhibition of autooxidation of pyrogallol [28]. Briefly, in a cuvette, 970 *μ*L of 50 mM Tris/1 mM EDTA buffer (pH 8.2), 4 *μ*L of catalase (CAT) (30 *μ*M), and 10 *μ*L of homogenate were added together and mixed. After that, 16 *μ*L of pyrogallol (24 mM in 10 mM HCl) was added and total SOD activity was determined at 36°C in a spectrophotometer (420 nm) for 120 s. Results were expressed in units of SOD per milligram of protein (U SOD·mg prot^−1^).

### 2.7. Hematological Analysis

After decapitation, blood was immediately collected into heparinized (30 IU·mL^−1^ final volume) tubes (for metabolite measurements) or in disodium EDTA-treated tubes (2 mg·mL^−1^ final volume). Hematological parameters were investigated in EDTA samples in a Horiba ABX Micros 60 hematology analyzer (for quantitative cell analysis) [24].

### 2.8. H-Index (eHSP72/iHSP70 Ratio)

Extracellular-to-intracellular HSP70 ratio index (H-index) has been described as a novel and overall index of the immunoinflammatory status of an individual. The rationale for H-index is that the higher the level of eHSP70 is, the greater the inflammatory signal is, due to the proinflammatory nature of the protein. Conversely, if cells are able to respond to stressful stimuli by enhancing iHSP70 production, they simultaneously enter a state of anti-inflammation. First, by definition, the eHSP70 and iHSP70 levels and the eHSP70/iHSP70 ratio in control groups (Rc) were considered as a baseline (Rc = 1.0). Thereafter the eHSP70/iHSP70 ratio in a stressful situation such as that in the experimental groups (Rexp) can be calculated as the quotient of different values relative to the Rc. Hence, the H-index (Rexp/Rc) allows comparisons between any stressful situation and the control [15, 17, 24].

### 2.9. Statistical Analyses

Statistical analysis was developed using two-way ANOVA with repeated measures followed by Tukey's post hoc test for differences in body weight, fasting glycaemia, glycaemia during GTT, and glycaemia during exercise. One-way ANOVA was used for comparison of IAUC results. Student's *t*-test was used to analyze all other variables. All statistical analyses were performed using GraphPad 7.0 for Windows. The level of significance was set to *P* < 0.05, and the results were expressed as mean ± Sd.

## 3. Results

**Figure 2:**
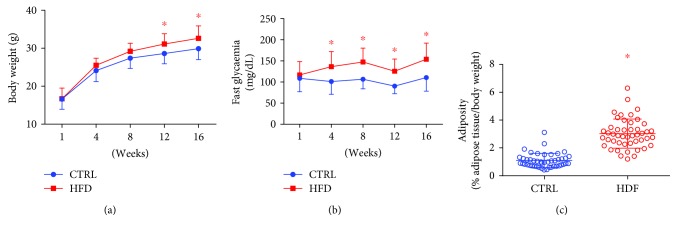
Effects of HFD consumption on body weight, fasting glycaemia, and adiposity in mice. Mice received standard chow (CTRL, *n* = 29) or high-fat diet (HFD, *n* = 31) over 16 weeks. HFD increased body weight (a), fasting glycaemia (b), and adiposity (c) of mice. Data are expressed as mean ± standard deviation. ^∗^*P* < 0.05 compared to the control. Two-way ANOVA with repeated measures followed by post hoc Tukey's test (a, b). ^∗^*P* < 0.05 vs. CTRL Student's *t*-test in (c).

**Figure 3:**
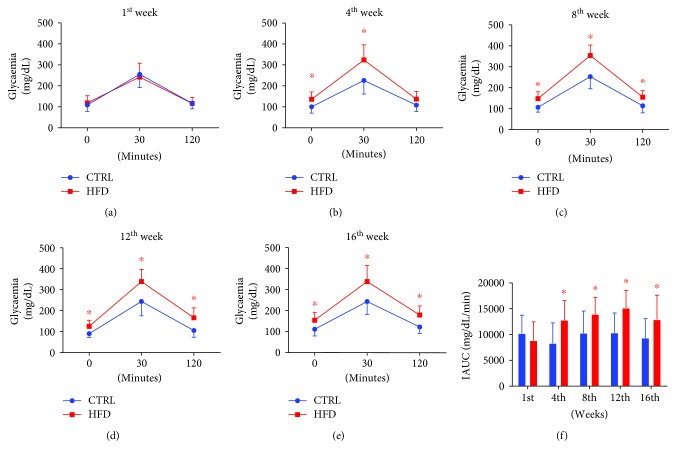
Effects of HFD on glucose tolerance test response in mice. Mice received standard chow (CTRL, *n* = 29) or high-fat diet (HFD, *n* = 31) over 16 weeks. Glucose tolerance test (GTT) was performed by administration (i.p.) of glucose (1 g/kg) before HFD intake in the first week (a), then following HFD in the 4th (b), 8th (c), 12th (d), and 16th (e) week. Glucose intolerance was confirmed based on the IAUC calculation (f). Data was expressed as mean ± standard deviation. ^∗^*P* < 0.05 vs. the CTRL group. Two-way ANOVA with repeated measures followed by post hoc Tukey's test (a–e) and one-way ANOVA followed by post-hoc Tukey's test (f).

At 30 days old, the mice were randomly separated in two groups, receiving the standard diet or HFD for 16 weeks. HFD treatment increased body weight, fasting glycaemia, and adiposity of mice compared to normally fed mice (Figure 2). Also, HFD mice presented with glucose intolerance via GTT, from the 4th week of HFD consumption (Figure 3).

**Figure 4:**
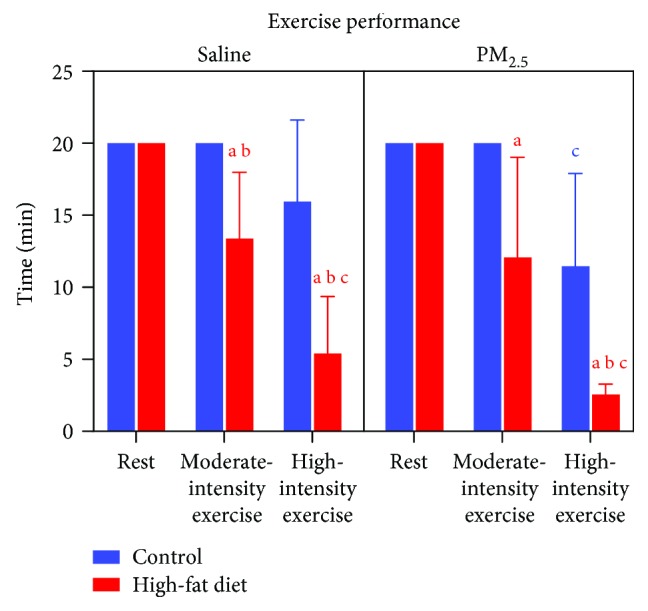
Effects of exercise under PM_2.5_ exposure on swim performance of HFD-treated mice. Mice received standard chow (CTRL, *n* = 29) or high-fat diet (HFD, *n* = 31) over 16 weeks. After animals received saline or PM_2.5_ by intranasal instillation, they were rested or submitted to exercise at moderate or high intensity. Data expressed was mean ± standard deviation. “a” means difference vs. the CTRL group in the same intensity, “b” means difference vs. the moderate-intensity group in the same diet treatment, and “c” means difference vs. the group that remained at rest in the same diet treatment. Two-way ANOVA *P* < 0.05.

At the end of the 16th week, mice received either saline or PM_2.5_ by intranasal instillation and were submitted to moderate- or high-intensity exercise or remained at rest. Rested mice were left for 20 min after saline or PM_2.5_ instillation (Figure 4), and we observed that mice that received normal chow were able to swim for 20 min with moderate intensity workload and without any effect from PM_2.5_ instillation (Figure 4). However, at moderate-intensity workload, HFD mice did not reach 20 minutes of exercise, performing only 13.3 ± 4.6 and 12.0 ± 6.9 minutes of exercise (HFD and HFD + PM_2.5_, respectively; see Figure 4). Also, all mice submitted to high-intensity exercise did not reach 20 minutes of exercise and HFD mice presented the lowest physical capacity in comparison to control mice in both situations: Without pollution, control mice swam for 15.9 ± 5.6 minutes, while HFD-treated mice swam for only 5.4 ± 3.6 minutes. Under PM_2.5_ exposure, control mice swam for 11.4 ± 6.4 minutes, while HFD-treated mice swam for only 2.5 ± 0.7 minutes (Figure 4).

**Figure 5:**
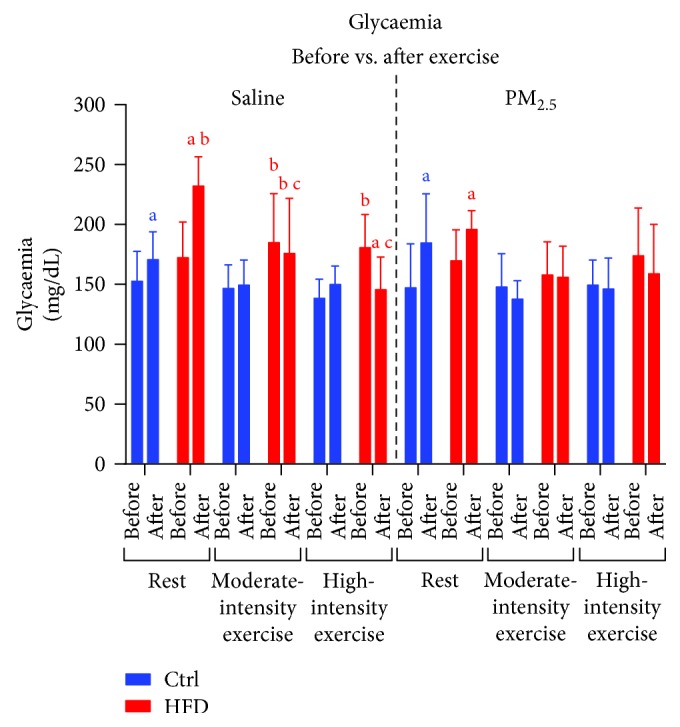
Effects of exercise under PM_2.5_ exposure on glycaemia of HFD-treated mice. Mice received standard chow (CTRL, *n* = 29) or high-fat diet (HFD, *n* = 31) over 16 weeks. After animals received saline or PM_2.5_ by intranasal instillation, they were rested or submitted to exercise at moderate or high intensity. Data expressed was mean ± standard deviation. “a” means statistical difference in the comparison before vs. after in the same group. “b” means the statistical difference in comparison with the CTRL group in the same intensity. “c” means the statistical difference in comparison with the moderate-intensity and rest groups in the same diet treatment. Two-way ANOVA, *P* < 0.05.

Mice remaining at rest in shallow water showed an increase in glycaemia, but it was higher in HFD mice than normal-chow mice (Figure 5). Moderate exercise did not modify the mice glycaemia, but the values in the HFD were above that of normal-chow mice, while the high-intensity exercise decreased the glycaemia in HFD mice (Figure 5). The PM_2.5_ inhalation had no influence in rested mice or those submitted to moderate exercise since the results were similar to that of the saline administration group (Figure 5). However, under PM_2.5_ exposure, HFD mice submitted to high-intensity exercise showed no decrease in glycaemia as that observed in the saline administration group (Figure 5).

**Figure 6:**
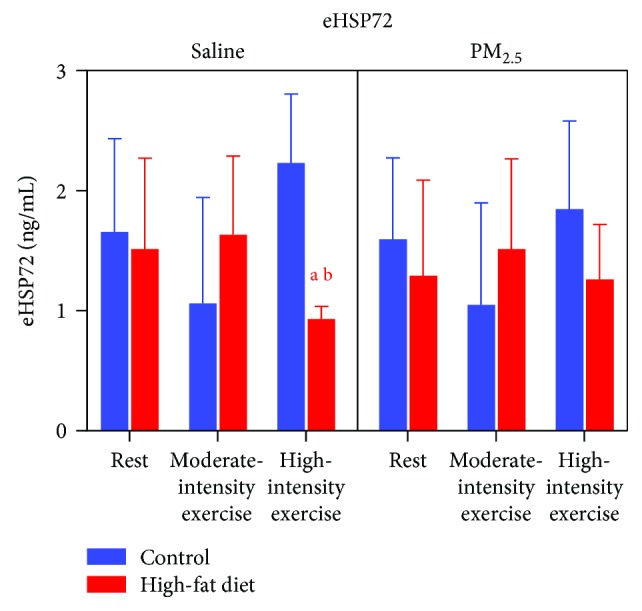
Effects of exercise under PM_2.5_ exposure on plasma eHSP70 levels of HFD-treated mice. Mice received standard chow (CTRL, *n* = 29) or high-fat diet (HFD, *n* = 31) over 16 weeks. After animals received saline or PM_2.5_ by intranasal instillation, they were rested or submitted to exercise at moderate or high intensity. Data expressed was mean ± standard deviation. “a” means the statistical difference in comparison with the CTRL group in the same intensity and “b” means the statistical difference in comparison with the moderate-intensity group in the same diet treatment. Two-way ANOVA, *P* < 0.05.

There was no difference in eHSP72 levels in HFD mice when compared to normal-chow mice at rest and when submitted to moderate exercise or upon exposure to PM_2.5_ (Figure 6). However, high-intensity exercise decreased eHSP72 levels in HFD mice but this effect was not observed in animals exposed to PM_2.5_ (Figure 6).

**Figure 7:**
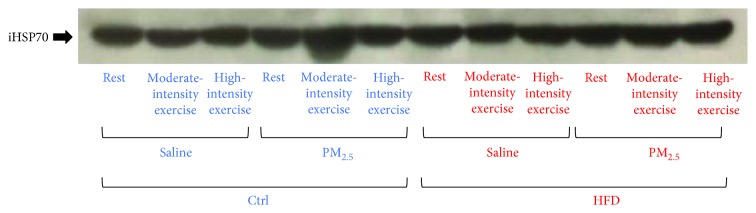
Effects of exercise under PM_2.5_ exposure on plasma iHSP70 lung levels of HFD-treated mice. Mice received standard chow (CTRL, *n* = 29) or high-fat diet (HFD, *n* = 31) over 16 weeks. After animals received saline or PM_2.5_ by intranasal instillation, they were rested or submitted to exercise at moderate or high intensity. This is a representative blot for HSP70 detection in mice lungs.

HFD increased iHSP70 levels in the lung of rested mice (Table 1, and the representative blot is in Figure 7). Lower levels of lipid peroxidation were observed in HFD mice submitted to moderate-intensity exercise in comparison with CTRL (Table 1). Also, high-intensity exercise decreased eHSP70/iHSP70 ratio levels (plasma/lung HSP70 ratio) in HFD mice but this effect was not observed in animals exposed to PM_2.5_ (Table 1). Total SOD antioxidant enzyme activity was not influenced in any experimental group (Table 1).

Changes in hematological parameters are shown in Table 2. The majority of parameters were not modified in our study. HFD induced an increase in lymphocyte count (vs. CTRL in resting groups). Moderate exercise increased the neutrophil count in HFD in comparison with CTRL animals, and high-intensity exercise increased red blood cell count and lymphocyte count in the HFD group (vs. CTRL in the same intensity).

## 4. Discussion

In our study, we studied the effects of HFD on subclinical and clinical parameters, in mice submitted to exercise under PM_2.5_ exposure. HFD mice presented an expected increase in body weight and adiposity, with an impaired glucose tolerance. This profile was accompanied by a poorer exercise performance, along with lower eHSP70 and eHSP70/iHSP70 ratio levels in comparison to the CTRL group. High-intensity exercise decreased glycaemia in HFD mice only and in the absence of PM_2.5_ exposure. The PM_2.5_ exposure promoted more hematological effects in HFD mice in comparison to CTRL, and this occurred in mice submitted to moderate- or high-intensity exercise. Thus, our study showed novelty in terms of the HSR as follows: (a) the influence of obesity/T2DM in eHSP70 plasma concentration and H-index (eHSP72/iHSP70 ratio) after exercise was dependent on exercise intensity, (b) acute environmental air pollution exposure modified the effects of exercise in obesity, and (c) these effects on HSP70 were not accompanied by altered oxidative stress biomarkers or by hematological changes.

The first step of our study was to induce obesity and glucose intolerance in B6._129_SF2/J mice by HFD. It was suggested that B6 mice used in our study should be more resistant to the effects of HFD due to decreased intestinal absorption of lipids, which would characterize the strain as less susceptible to the effects of HFD [29]. In the study of Bock et al. [29], lower responsiveness to GTT and alterations in fasting glucose were observed after four weeks of HFD intake in adult animals. In contrast, in the present study, we exposed the animals to HDF after weaning (four weeks old). The data presented herein, confirm previous findings, were the effects of HFD following weaning result in a greater adiposity and metabolic disorder profile, and therefore, this experimental design can be considered suitable for the of obesity [24]. In addition to the promotion of obesity (increased body mass and adipose tissue), this experimental protocol has also been used for the study of chronic and low-grade inflammation, and altered HSP70 expression [15, 16].

The second step of our study was to submit HFD and CTRL mice to 50 *μ*g of PM_2.5_ exposure (or saline). A single administration of particle suspension into the nostril of mice is aimed at simulating an acute exposure to an environment similar to that proposed by interim target 2 from the World Health Organization 24-hour concentration air quality guidelines. Based on published risk coefficients from multicenter studies and meta-analyses, urban PM_2.5_ concentration of 50 *μ*g/m^3^ represents about 2.5% increase of short-term mortality than 25 *μ*g/m^3^ PM_2.5_ concentration; limits are proposed by the Air Quality Guideline [4]. A previous study used the same dose to represent a particle concentration of 29 *μ*g/m^3^ in an urban area, which is the value found in a polluted city [30]. Thus, our protocol was elaborated to simulate closer a “real-world” exposure study (because of the dose, the source—urban area of Sao Paulo, Brazil—and the complex mix of metal adsorbed in the particles) than a more specifically toxicological study, evaluating the effect of each component of particle.

It is important to highlight that the majority of experimental studies regarding adverse effects of PM_2.5_ on oxidative stress parameters (and others) are conducted with animals under rest conditions and use higher levels of particle exposure. Usually, oxidative stress is observed in experimental designs that expose mice or rats to high levels of concentrated particles [12], higher levels of aerosol suspension, or intratracheal particle instillation [25, 31]. In the same strain of mice used herein (B6_129_SF2/J) and in a similar PM_2.5_ exposure protocol (intranasal instillation of 5 *μ*g PM_2.5_, daily for 12 weeks), no increase in oxidative stress was observed in the PM_2.5_ exposure group [15]. Considering that upper airway filtration in mice can prevent up to 50% of particle deposition in alveolar spaces, the dose of 50 *μ*g of PM_2.5_ used herein represents a low-to-moderate level of environmental air pollution exposure. During exercise, breath frequency (n·min^−1^) and minute ventilation (mL·min^−1^) increased and were dependent on exercise intensity and also possibly on particle deposition within the lungs [32]. In comparison to rest conditions, exercise can increase particle deposition up to 6.0-fold in rodents [33]. However, even at high levels of particles, one exposure alone may not induce clinical effects after exercise, but only subclinical effects [31, 34]. In our study, PM_2.5_ exposure increased leukocytosis induced by exercise in HFD mice and this may represent a proinflammatory predisposition profile in HFD mice [35].

After PM_2.5_ instillation (or saline), the mice were submitted to one bout of moderate- or high-intensity exercise or remained at rest. Our high-intensity exercise protocol was performed by the attachment of an overload weight on the tail (8% of body weight) during the swimming exercise session. The fatigability of this protocol was previously tested in mice and rats with this workload. Indeed, this swimming time (20 min) was chosen because this is the time limit within which an untrained animal really swims before learning how to perform bobbing, which is a survival strategy used to conserve energy without doing exercise. On the other hand, with 4% body weight attached to the tail, mice can swim for 60 minutes or more. For this reason, the workloads used herein characterized adequately two distinct exercise intensities. It may be questioned whether the effects observed in the high-intensity exercise groups were induced by higher total energy expended during exercise than those observed in the moderate-intensity exercise groups. However, a moderate-intensity range of 60–75% of VO_2_max at 4.0–4.6% workloads has been suggested for swimming mice [26]. Additionally, an 8% workload may represent a high-intensity swimming exercise estimated as representing more than 90% of VO_2_max [36]. Despite this, the estimated total energy expended by animals from the high-intensity exercise groups (*x* min × 8% workload) was similar to that from the moderate-intensity groups (*x* min × 4% workload). Assuming 4.8 kcal (20 kJ) of energy equivalent of consumed O_2_ and 100 mL·kg^−1^·min^−1^ as mouse VO_2_max [26, 37], the total energy expended in each exercise session for the moderate intensity groups was between 0.08 and 0.18 kcal (assuming 10–20 min × 0.025 kg × 60–75 mL·kg^−1^·min^−1^), while in the high-intensity exercise groups, it was 0.05–0.16.5 kcal (considering 5–15 min × 0.025 kg × 90 mL·kg^−1^·min^−1^). Since HFD mice presented low VO_2max_ [37], the performance of HFD mice in the swimming test was observed in our study during one swimming exercise session, in both moderate- and high-intensity exercise.

In our study, we observed that rested mice increased glycaemia and that this effect was more pronounced in HFD mice. Also, the decrease in glycaemia after high-intensity exercise in HFD mice was not observed under PM_2.5_ exposure in HFD mice. These results may be explained by the effects of swimming exercise, HFD, and PM_2.5_ on the autonomic nervous system: first, swimming performance in the rodents is known to be dramatically influenced by hot or cold water temperature generating early fatigue [26]. Although the water temperature range chosen herein (31 ± 1°C) has been suggested to be the optimal water temperature to exercise, animals that remained in rest have different responses than exercising animals in the same water temperature [17], which suggests a difference in the sympathetic stimulation and vascular response in these situations. Added to this, HFD mice presents a reduced vascular adrenergic contractility [38]. This situation may evoke an overstimulation of the sympathetic nervous system that was more evident with the HFD mice. Second, the catecholamine response to exercise may be blunted in obese/diabetic subjects, presumably indicating autonomic dysfunction during moderate-intensity exercise, resulting in mild hyperglycaemia, associated with defects in hepatic glucokinase activity [39]. Finally, particle inhalation may cause autonomic nervous system imbalance [11, 12], and, although not causing hemodynamic altered responses during exercise [31], the PM_2.5_ effects may be influenced by early vascular inflammation and endothelial dysfunction observed in HFD mice that present reduced nitric oxide production-impaired insulin signal [40]. In this way, PM_2.5_ exposure is associated with increases in systemic cytokines as TNF-*α* and IL-6 levels, evoking a pronounced pulmonary and systemic inflammatory response [41] that in our work may be a reason for altered exercise performance and glycaemia response in HFD mice.

After the exercise session, mice were euthanized and eHSP70 and eHSP70/iHSP70 levels were determined. Under stress conditions, cells from different tissues increase iHSP70 expression (cellular stress response) and also export this protein to the circulation [42]. High plasma levels of eHSP70 are correlated with energetic balance impairment, alteration of pro-/anti-inflammatory status, and redox homeostasis [15, 42–44]. On the other hand, absence or inhibition of HSP70 expression is associated with increased cell vulnerability and decreased ability to cope with stress [45], which may promote apoptosis [46]. In our study, we observed an increase in lung iHSP70 levels in HFD mice when compared to control and this may indicate that the lungs are under stress induced by HFD but they were still able to maintain the HSR, essential for lung protection against oxidative stress induced by PM_2.5_. In other tissues such as muscle, low iHSP70 levels were observed in animals chronically exposed to the HFD intake [16]. A similar profile was also observed in the liver and adipose tissue [47]. This decrease in iHSP70 was correlated with glucose intolerance and insulin resistance in obese mice [16]. This defect in HSR, as commonly observed in chronic cases of inflammation, has been associated with many obesity-related diseases and dysfunctions including insulin resistance, T2DM, and nonalcoholic hepatic steatosis [47, 48]. In the other side, eHSP72 is related to immune system activation. Indeed, eHSP72 has been reported as an inductor of different immune cell activations attributed to its known capacity to bind to Toll-like receptors 2 and 4 (TLR2 and TLR4) [19, 20]. However, assuming that exercise, a known inducer of eHSP72 release [17, 18, 20, 23, 49], induces an anti-inflammatory response, each single bout of exercise may induce an acute activation of inflammatory response followed by a membrane downregulation of Toll-like receptors resulting in a posterior anti-inflammatory response. Thus, the lower capacity to release eHSP72 of HFD mice under high-intensity exercise could result in a higher cellular response to inflammatory mediators in long term, worsening glucose unbalance. In this way, our study showed that a lower concentration of plasma eHSP70 was present in HFD mice submitted to high-intensity exercise, in comparison to CTRL mice. Since HFD mice were not able to swim for 20 minutes, the lower eHSP70 levels in plasma may represent an insufficient accumulation of exercise effort (time × load). These data are in agreement with the hypothesis that a minimum amount of exercise effort is necessary to promote health effects [50]. Thus, the minimum of level physical activity may not be reached due early fatigue and this represents a key limitation in the obese condition.

In our study, the glycaemia and eHSP70 levels decreased in the HFD group after high-intensity exercise and this effect was not observed in other groups (moderate-intensity exercise or PM_2.5_ groups). It was demonstrated that eHSP70 increased throughout exercise and was attenuated by glucose ingestion, mainly by inhibition of hepatosplanchnic eHSP70 release [51]. Thus, the lower levels of eHSP70 in HFD submitted to exercise can be possibly explained by the hyperglycaemic state of mice. Also, in the presence of PM_2.5_, the control of eHSP70 release may be related to glycaemia which may also be affected by liver stress induced by PM_2.5_. In this way, PM_2.5_ exposure induces endoplasmic reticulum stress in the lung and liver [52]. Endoplasmic reticulum stress, also called the unfolded protein response (UPR), is an intracellular stress signaling cascade that protects cells from stress caused by the accumulation of unfolded or misfolded proteins and is very sensitive to changes of intracellular homeostasis. Physiological states that increase protein folding demand or stimulate the disruption of protein folding reactions create an imbalance between the protein folding load and capacity of the endoplasmic reticulum. The UPR is related to many alterations in heat shock protein families, including the HSP70 family, and is associated with diabetic complications [53].

Due to the versatility of HSP70 to induce different responses related to inflammation according to its location, it is proposed that this protein may represent an important marker for the immunoinflammatory state during exercise [17, 20]. Also, HSP70 balance measured by mathematical calculation of the H-index as reported in other studies [15, 17, 23, 24] may represent an important biomarker of the health/disease process, as well as serve as a reference in subclinical biological processes that occur in the body. In other words, it is expected that acute exercise bouts signal a “stressful situation” to all physiological systems [20], leading to transient but augmented eHSP70 plasma levels, and regular exercise training may lead to an overall decrease in eHSP70 levels [49], as a “heat shock tolerance” phenomenon or exercise-induced stress adaptation. These effects support the prescription of exercise as a tool to decrease or maintain the normal eHSP70 and eHSP70/iHSP70 ratio values and, consequently, to promote optimum glucose metabolism. It is also well known that during physical exercise, IL-6 can be expressed and released by the skeletal muscle, and within the extracellular space, it binds to the IL-6 receptor in an autocrine action. Interestingly, the “myokine” IL-6 has also been found to induce HSF-1 translocation to the nucleus upregulating heat-induced HSP70 gene and protein expression. Since the anti-inflammatory response to acute exercise is attributed to increased circulating levels of known anti-inflammatory cytokines that are dependent on exercise effort [49, 54], our data suggested that as obese subjects find it difficult to reach adequate levels of exercise, this may also cause an impairment in appropriate adaptive HSR responses and subsequent metabolic benefits to obese/T2DM subjects.

## 5. Conclusion

Our study showed that HFD impaired exercise performance and weakened the standard heat shock response to exercise, as observed by lower levels of eHSP70 and extra-to-intracellular HSP70 ratio levels. PM_2.5_ exposure modified glycaemic response to exercise and altered hematological responses in HFD mice. Our data indicated that obesity is a critical health condition for exercise prescription under PM_2.5_ exposure.

## Figures and Tables

**Table 1:** Effects of exercise under PM_2.5_ exposure on lung oxidative stress, iHSP70 levels, and eHSP70/iHSP70 ratio levels of HFD-treated mice.

	Rest			Moderate-intensity exercise			High-intensity exercise		
	Control	HFD	*t*-test	Control	HFD	*t*-test	Control	HFD	*t*-test

TBARS	0.18 ± 0.06	0.13 ± 0.03	*P* = 0.112	0.32 ± 0.10	0.14 ± 0.02	*P* = 0.0004^∗^	0.17 ± 0.02	0.14 ± 0.04	*P* = 0.134
SOD	0.17 ± 0.01	0.19 ± 0.02	*P* = 0.068	0.19 ± 0.02	0.19 ± 0.02	*P* = 0.874	0.20 ± 0.02	0.19 ± 0.03	*P* = 0.562
iHSP70	1.02 ± 0.02	1.27 ± 0.14^∗^	^∗^ *P* = 0.034	1.26 ± 0.27	1.29 ± 0.14	*P* = 0.861	1.08 ± 0.09	1.36 ± 0.24	*P* = 0.129
eHSP70/iHSP70 ratio	1.00 ± 0.31	0.65 ± 0.32	*P* = 0.248	0.94 ± 0.76	0.67 ± 0.39	*P* = 0.608	1.40 ± 0.47	0.42 ± 0.09^∗^	^∗^ *P* = 0.023

	Rest + PM_2.5_			Moderate Intensity Exercise + PM_2.5_			High-Intensity Exercise + PM_2.5_		
	Control	HFD	*t*-test	Control	HFD	*t*-test	Control	HFD	*t*-test

TBARS	0.33 ± 0.14	0.13 ± 0.02^∗^	*P* = 0.003^∗^	0.29 ± 0.07	0.12 ± 0.02^∗^	*P* = 0.0001^∗^	0.14 ± 0.02	0.16 ± 0.06	*P* = 0.670
SOD	0.20 ± 0.01	0.19 ± 0.03	*P* = 0.417	0.17 ± 0.02	0.19 ± 0.04	*P* = 0.401	0.17 ± 0.02	0.18 ± 0.03	*P* = 0.378
iHSP70	1.15 ± 0.05	1.45 ± 0.26	*P* = 0.118	1.30 ± 0.32	1.48 ± 0.19	*P* = 0.447	1.27 ± 0.12	1.20 ± 0.17	*P* = 0.587
eHSP70/iHSP70 ratio	0.76 ± 0.28	0.36 ± 0.09	*P* = 0.074	0.33 ± 0.18	0.65 ± 0.33	*P* = 0.211	0.52 ± 0.06	0.81 ± 0.22	*P* = 0.089

**Table 2:** Effects of exercise under PM_2.5_ exposure on the hematological profile of HFD-treated mice.

	Rest			Moderate-intensity exercise			High-intensity exercise		
	Control (*n* = 5)	HFD (*n* = 7)	*t*-test	Control (*n* = 7)	HFD (*n* = 8)	*t*-test	Control (*n* = 8)	HFD (*n* = 8)	*t*-test

RBC (10^6^/mm^3^)	8.4 ± 0.5	8.3 ± 1.0	*0.842*	8.0 ± 1.0	8.1 ± 0.9	*0.841*	8.4 ± 0.7	8.0 ± 1.2	*0.429*
HGB (g/dL)	13.8 ± 1.5	13.2 ± 1.3	*0.475*	12.4 ± 2.4	12.6 ± 1.0	*0.832*	13.8 ± 1.1	12.3 ± 2.1	*0.095*
HCT (%)	37.0 ± 3.2	36.6 ± 4.5	0.868	34.7 ± 4.6	35.6 ± 4.2	*0.698*	37.2 ± 3.2	35.1 ± 5.9	*0.391*
PLT (10^3^/mm^3^)	958 ± 120	896 ± 256	*0.628*	831 ± 154	879 ± 144	*0.543*	889 ± 191	820 ± 283	0.576
Neutr (10^3^/mm^3^)	0.32 ± 0.09	0.47 ± 0.16	*0.089*	0.28 ± 0.19	0.31 ± 0.19	*0.765*	0.36 ± 0.10	0.35 ± 0.26	0.920
Monoc (10^3^/mm^3^)	0.27 ± 0.07	0.39 ± 0.14	*0.110*	0.28 ± 0.06	0.27 ± 0.19	*0.896*	0.32 ± 0.11	0.31 ± 0.16	0.886
Lymph (10^3^/mm^3^)	5.18 ± 0.56	7.64 ± 1.93	*0.021 * ^∗^	6.94 ± 2.02	8.50 ± 1.9	*0.147*	7.59 ± 1.72	8.0 ± 2.87	0.734
Neutr/lymph	0.05 ± 0.01	0.056 ± 0.016	*0.478*	0.054 ± 0.02	0.038 ± 0.02	*0.146*	0.043 ± 0.019	0.043 ± 0.02	0.999

	Rest + PM_2.5_			Moderate-intensity exercise + PM_2.5_			High-intensity exercise + PM_2.5_		
	Control (*n* = 7)	HFD (*n* = 7)	*t*-test	Control (*n* = 5)	HFD (*n* = 7)	*t*-test	Control (*n* = 8)	HFD (*n* = 8)	*t*-test

RBC (10^6^/mm^3^)	8.1 ± 1.3	8.3 ± 0.7	*0.726*	8.6 ± 0.4	8.0 ± 0.9	*0.197*	7.2 ± 2.1	9.2 ± 1.2	*0.034 * ^∗^
HGB (g/dL)	13.2 ± 2.2	13.2 ± 1.1	*0.999*	13.7 ± 1.7	12.7 ± 1.6	*0.322*	12.2 ± 3.2	14.0 ± 2.5	*0.230*
HCT (%)	35.7 ± 4.9	36.1 ± 2.8	*0.854*	37.8 ± 3.3	35.6 ± 3.6	*0.306*	36.7 ± 3.1	39.1 ± 5.9	*0.325*
PLT (10^3^/mm^3^)	807 ± 128	897 ± 140	*0.233*	934 ± 85	924 ± 100	*0.859*	960 ± 174	875 ± 150	*0.313*
Neutr (10^3^/mm^3^)	0.285 ± 0.073	0.390 ± 0.20	*0.216*	0.336 ± 0.183	0.540 ± 0.105	*0.033 * ^∗^	0.348 ± 0.145	0.443 ± 0.327	*0.465*
Monoc (10^3^/mm^3^)	0.228 ± 0.083	0.315 ± 0.121	*0.142*	0.279 ± 0.115	0.388 ± 0.136	*0.176*	0.292 ± 0.130	0.418 ± 0.129	*0.072*
Lymph (10^3^/mm^3^)	5.922 ± 1.84	7.614 ± 2.67	*0.192*	8.091 ± 0.93	9.72 ± 2.29	*0.167*	5.874 ± 3.28	10.764 ± 2.74	*0.006 * ^∗^
Neutr/lymph	0.055 ± 0.018	0.055 ± 0.024	*0.999*	0.043 ± 0.020	0.045 ± 0.016	*0.850*	0.060 ± 0.020	0.0375 ± 0.020	*0.037 * ^∗^

## Data Availability

The data used to support the findings of this study are available from the corresponding author upon request.

## Abbreviations

AUC: Area under the curve
eHSP70: Extracellular 70 kDa heat shock proteins
GTT: Glucose tolerance test
HFD: High-fat diet
HSP70: 70 kDa heat shock proteins
H index: eHSP70/iHSP70 ratio
iHSP70: Intracellular 70 kDa heat shock proteins
PM_2.5_: Fine particulate matter
T2DM: Type 2 diabetes.
